# Tolerability and safety of artesunate-amodiaquine and artemether-lumefantrine fixed dose combinations for the treatment of uncomplicated *Plasmodium falciparum* malaria: two open-label, randomized trials in Nimba County, Liberia

**DOI:** 10.1186/1475-2875-12-250

**Published:** 2013-07-17

**Authors:** Birgit Schramm, Parastou Valeh, Elisabeth Baudin, Charles S Mazinda, Richard Smith, Loretxu Pinoges, Timothy Sundaygar, Yah M Zolia, Joel J Jones, Eric Comte, Arnaud Bruneel, Michel Branger, Vincent Jullien, Gwenaelle Carn, Jean-René Kiechel, Elizabeth A Ashley, Philippe J Guérin

**Affiliations:** 1 Epicentre, 75011 Paris, France; 2 National Malaria Control Programme, Ministry of Health and Social Welfare, Monrovia, Liberia; 3 Médecins Sans Frontières, 1211 Geneva, Switzerland; 4 AP-HP, Biochimie Métabolique et Cellulaire, Hôpital Bichat, 75018 Paris, France; 5 Service de Virologie, Centre Hospitalier Bichat-Claude Bernard, 75018 Paris, France; 6 INSERM U663, Université Paris Descartes, 75006 Paris, France; 7 Drugs for Neglected Diseases initiative, 1202 Geneva, Switzerland; 8 Centre for Tropical Medicine, Nuffield Department of Clinical Medicine, University of Oxford, CCVTM, Oxford, UK

**Keywords:** Malaria, Artemisinin, Tolerability, Randomized trial, Liberia

## Abstract

**Background:**

Safety surveillance of widely used artemisinin-based combination therapy (ACT) is essential, but tolerability data in the over five years age group are largely anecdotal.

**Methods:**

Two open-label, randomized trials were conducted in Nimba County, Liberia: i) the main tolerability trial with 1,000 *Plasmodium falciparum* malaria patients aged over five years (Study-T), and, ii) an efficacy trial with a secondary objective of collecting tolerability data among 300 children age six to 59 months (Study-E). In both studies patients were randomized to fixed-dose artesunate-amodiaquine (ASAQ Winthrop®) or artemether-lumefantrine (AL, Coartem®), respectively. Clinical- and laboratory-adverse events (AEs) were recorded until day 28.

**Results:**

Study-T: most patients experienced at least one AE. Severe AEs were few, primarily asymptomatic blood system disorders or increased liver enzyme values. No treatment or study discontinuation occurred. Mild or moderate fatigue (39.8% *vs* 16.3%, p < 0.001), vomiting (7.1% *vs* 1.6%, p < 0.001), nausea (3.2% *vs* 1.0%, p = 0.01), and anaemia (14.9% *vs* 9.8%, p = 0.01) were more frequently recorded in the ASAQ *versus* AL arm. Study-E: mild or moderate AEs were common, including anaemia, fatigue, vomiting or diarrhoea. The few severe events were asymptomatic blood system disorders and four clinical events (pneumonia, malaria, vomiting and stomatitis).

**Conclusion:**

Both ASAQ and AL were well tolerated in patients of all age groups. No unexpected AEs occurred. Certain mild or moderate AEs were more frequent in the ASAQ arm. Standardised safety surveillance should continue for all forms of ACT.

**Trial registration:**

The protocols were registered with Current Controlled Trials, under the identifier numbers ISRCTN40020296, ISRCTN51688713, (http://www.controlled-trials.com).

## Background

Artemisinin-based combination therapy (ACT) is a key tool in malaria control and is the World Health Organization’s (WHO) recommended treatment for uncomplicated *Plasmodium falciparum* malaria. Among the most widely available current ACT are artemether-lumefantrine (AL) and artesunate-amodiaquine (AS + AQ). Artemisinin and its derivatives are well tolerated [1], and the combination treatments AS + AQ and AL are considered safe and efficacious [2-4]. Serious safety issues were noted in the past in association with AQ administered alone at high-dose for treatment or long-term prophylaxis, with case reports of severe adverse events (AEs) of agranulocytosis, hepatitis [5-7], or severe neurotoxicities (involuntary movements/dystonia) [8]. A dose-dependent risk for neutropaenia was recently reported for seven-day artesunate monotherapy (at higher than usual dose: 6 mg/kg/day) in adult malaria patients [9]. A recent review discussed case reports of delayed hemolytic anemia following treatment of severe malaria with artemisinin-derivates, but the data were considered insufficient to conclude on an association with artesunate treatment [10]. Hemolytic anemia is also known to occur as a consequence of malaria infection itself.

Ideally ACT should be provided as fixed-dose combinations (FDC) to improve compliance. The first FDC of AS with AQ, (ASAQ Winthrop® Sanofi-Aventis) achieved WHO-prequalification in 2008 and is now registered in 32 countries. Phase III studies showed safety, good tolerability and high efficacy [4,11,12]. Gathering information on the safety and tolerability of new products after registration can be difficult, as pharmacovigilance systems are often weak or non-existent. The present study aimed to provide comprehensive information on the clinical and laboratory tolerability profile of the new ASAQ FDC in a large group of patients aged six years and older who are studied less frequently and, unlike younger patients, able to express symptoms. Complementary safety and tolerability data are also reported here from a parallel efficacy study on ASAQ and AL in children under five years [13].

## Methods

### Study site, objectives and design

Two open-label, randomized, controlled, two-arm clinical trials were conducted in the Comprehensive Healthcare Center (CHC) of Saclepea, Nimba County, Northern Liberia. Médecins sans Frontières (MSF) Switzerland coordinated the CHC in collaboration with the Ministry of Health. Since 2004, AS + AQ co-blister has been provided as first-line treatment in this area.

#### Tolerability trial in over five year old patients (Study-T)

The principal objective of the main study reported here was to describe the tolerability of the fixed dose of ASAQ (Winthrop FDC) in adults and children aged over five years with uncomplicated *P*. *falciparum* malaria compared to artemether-lumefantrine (AL) (tolerability trial, Study-T). Tolerability was assessed based on the frequency and severity of adverse events. Inclusion criteria were: age older than five years; weight equal or higher than 18 kg; fever (axillary temperature ≥ 37.5°C), or history of fever in previous 48 hours; microscopic confirmation of asexual stages of *P*. *falciparum* or mixed infection; high probability of attending the follow-up visits; signed informed consent (responsible caregiver). Exclusion criteria were: pregnancy; or severe malaria; severe anaemia (<5 g/dl haemoglobin (Hb)); or full course of AS + AQ or AL treatment or more than two doses of another anti-malarial in the past four weeks; or known hypersensitivity to artemisinin derivates, amodiaquine, or artemether-lumefantrine; or concomitant febrile illness if additional medication is required other than antipyretics.

#### Efficacy trial in under five year old patients (Study-E)

The principal objective of the second study was to assess the genotyping-adjusted cure rates of ASAQ compared to AL in 300 children six to 59 months old with uncomplicated *P*. *falciparum* malaria after 42 days of follow up (efficacy trial, Study-E) [13]. As a secondary objective the safety and tolerability of ASAQ and AL was assessed up to day 28. Inclusion criteria were: age six to 59 months; fever (axillary temperature ≥ 37.5°C) or fever or history of fever in the previous 48 hours; blood smear-confirmed asexual stages of *P*. *falciparum* malaria (*P*. *falciparum* mono-infection) and parasite density between 2000–200,000/μl blood; high probability of attending follow-up; signed informed consent (responsible caregiver). Exclusion criteria were: general danger signs; severe/complicated malaria [14]; severe anaemia (<5 g/dl haemoglobin); full course of the treatments under study in past 10 days; known hypersensitivity to the study drugs; concomitant febrile illness other than malaria that may confound outcome; severe malnutrition (weight-for-height < 70% of median and/or symmetrical edema involving at least the feet).

### Randomization and blinding

Between September 2008 and May 2009, suspected malaria patients were pre-screened by an HRP-2 rapid diagnostic test (Paracheck®), followed by screening with a clinical examination performed by trained physician assistants, malaria blood smear, Hb from capillary blood (HemoCue®), and urine-pregnancy test (females ≥12 years). Patients who met the eligibility criteria of the respective trials were randomized to ASAQ or AL (ratio 1:1). In Study-T, randomization was stratified by weight (≤36 kg; >36 kg) to balance treatment allocation between adults and children. Allocation lists were computer-generated with a block size of six. The study site was unaware of block size. Treatment allocation was concealed in sealed opaque envelopes to be opened by study nurses in consecutive order at randomization. This was not disclosed to the medical staff performing the clinical assessments. The laboratory team was aware of treatment allocation since samples were taken to measure drug concentrations of the artemisinin partner compounds on day 0 and day 7, which required distinction between treatment arms.

### Ethics

The procedures followed were in accordance with the ethical standards of the Helsinki Declaration. All participants or responsible caretakers (≥18 years) gave written informed consent. The Liberian Institute for Biomedical Research (LIBR) ethics committee, the Ministry of Health and Social Welfare, Monrovia, Liberia, and the Comité de Protection des Personnes (CPP) Ile de France XI (Saint Germain en Laye), France, approved the studies. The studies were registered at Controlled Trials (http://www.controlled-trials.com/. ISRCTN51688713, ISRCTN40020296).

### Treatment

Both treatments were three-day oral regimens. ASAQ Winthrop® was one dose per day without co-administration of food, and AL (Coartem®, Novartis) was two doses per day, six to 12 hours between doses, administered with a high-fat cookie or breast-feeding encouraged. Dosage was by weight: ASAQ Winthrop® 5 to <9 kg: one tablet/day of both AS 25 mg/AQ 67.5 mg; 9 to <18 kg: one tablet/day of both AS 50 mg/AQ 135 mg; 18 to 36 kg: one tablet/day of both AS 100 mg/AQ 270 mg; ≥ 36 kg: two tablets/day of both AS 100 mg/AQ 270 mg. Coartem® tablet strength was 20 mg artemether/120 mg lumefantrine: 5 to <15 kg: one tablet/dose; 15 to <25 kg: two tablets/dose; 25 to <35 kg: three tablets/dose; ≥35 kg: four tablets/dose. All doses were administered in the study site followed by 30 minutes’ observation. If a dose was vomited/spat-out within 30 minutes, a full dose was re-administered. If the re-administered dose was vomited/spat-out within 30 minutes, the patient was withdrawn and rescue treatment given (parenteral quinine or intramuscular artemether).

### Clinical monitoring and laboratory follow up

At each visit (Study-T: days 0, 1, 2, 7, 28; Study-E: days 0, 1, 2, 3, 7, 14, 21, 28, 35, 42) a standardized symptoms questionnaire and physical examination were conducted. A β-human chorionic gonadotropin (βHCG) urine-pregnancy test was done on day 0 and 28 for females ≥12 years. In Study-T, malaria blood smears were obligatory on days 0, 2 and 28. In Study-E blood smears were done on days 0, 2, 3, 7, 14, 21, 28, 35, 42. On day 0 a serum sample (from venous blood) was stored at −20°C for further analyses. The routine assessment of blood levels of liver enzymes aspartate aminotransferase (AST) and alanine aminotransferase (ALT) was implemented after the start of the tolerability trial (Study-T), following the recommendation of the data safety monitoring committee. Liver function tests (LFTs) were conducted by assessment of blood levels of AST and ALT (Reflotron plus®, Roche Diagnostics) on day 0 (fresh serum) and on day 28 (finger-prick capillary blood). Missing day 0 AST values (Patients 1 to 306, analyser implemented after Study-T start) were measured retrospectively from frozen serum (by Modular D/P, Roche Diagnostics) and added to summary statistics (ALT not sufficiently stable in frozen serum). Baseline total bilirubin (direct) and creatinine were assessed retrospectively from day 0 serum by spectrophotometry (Modular D/P, Roche Diagnostics). Hb was measured on each visit (finger-prick capillary blood, HemoCue®). A full blood count (FBC) was done on days 0, 7 and 28 (Act5diff, Beckman Coulter®), or if the HemoCue® indicated anaemia.

### Anti-malarial blood concentrations

In both studies, day 0 and day 7 blood concentrations of AQ, desethyl-amodiaquine (DEAQ) (ASAQ arm) and lumefantrine (LF) (AL arm) were measured from dried spots of venous blood on filter paper using high performance liquid chromatography with ultraviolet or by tandem-mass spectrometry detection method, respectively [15,16]. Limits of quantification (LOQ) were <5 ng/ml for AQ or DEAQ, and <200 ng/ml for LF. Day 7 samples collected before day 6 or after day 8 were excluded from statistical analysis on blood concentrations. Summary statistics were provided on the safety population. The median day 7 blood concentrations were compared between patients with selected AEs of interest and patients without the respective AEs. These AEs were: fatigue (pooled AEs of fatigue, asthenia or weakness), vomiting, nausea, anaemia, or hepatotoxicity (pooled AEs of jaundice, hepatitis, hepatomegaly, AST and/or ALT) by two-sample Wilcoxon rank-sum (Mann–Whitney) test.

### Viral hepatitis serology

Baseline serum samples from both studies were retrospectively subjected to a screen for viral hepatitis serological markers: a) hepatitis B virus (HBV): hepatitis B surface antigen (HBs Ag) (Elisa, DiaSorin, Italy); b) if Ag HBs-positive a test for the presence of Immunoglobulin-anti-bodies against the hepatitis B core antigen (HBc IgM) was performed (1:10 dilution; Elisa, DiaSorin); c) hepatitis C virus (HCV): a test for the presence of immunoglobulin G anti-bodies to HCV(anti HCV IgG) (Elisa, Ingen); and, d) for hepatitis E virus (HEV): the serum of all patients with elevated baseline AST or ALT (values 2x upper limit normal range at any point during study participation) were tested for the presence of IgM antibodies to HEV (anti HEV IgM) (Elisa, Dia.Pro).

### Sample size

Study-T: one thousand participants allowed to detect a minimum of 2.7-3.3% difference between relatively low AE frequencies (1-2% in one study arm, versus 3.7-5.3% in the other study arm), and a minimum of 8–8.4% differences for relatively high AE frequencies (25-30% in one study arm versus 38.4% in the second arm (80% power and 0.05 significance level) (Power Analysis and Sample Size calculation Software for Windows, 2007, NCSS, Utah, USA). The Study-E sample size was powered to assess the non-inferiority of ASAQ *versus* AL treatment efficacy [13].

### Recording of adverse events and statistical analysis

Clinical- or laboratory signs and symptoms which occurred or worsened at any time after the first drug intake up to day 28 were recorded as adverse events (AEs) in both studies. AEs were defined according to International Conference on Harmonisation (ICH) guidelines for good clinical practice [17]. For each AE, onset date, severity grade, relationship to study medication (“definitely unrelated”, “unlikely related” or “possibly related”), seriousness (“serious” or “not serious”), outcome (“completely recovered”, “ongoing”, “death”, “unknown”, “recovered with sequelae”), and date were recorded. The severity of clinical events was graded using the common toxicity criteria (CTC) AE grading manual (version 3) [18], and laboratory events with the laboratory section of the Division of Microbiology and Infectious Diseases (DMID) severity grading scheme [19]; ≥12 years: adult toxicity table (May 2011), <12 years: paediatric toxicity table (February 2003)), applying the following overall severity grading: grade 1 (mild) = awareness of sign/symptom, easily tolerated; grade 2 (moderate) = discomfort enough to cause interference with usual activity; grade 3 (severe) = incapacitating with inability to work/perform usual activity; grade 4 = life threatening/or disabling. All randomized patients exposed to at least one dose of study drug were analysed (safety population). Before analysis, AEs were coded into standardized preferred terms (PT) using the Medical Dictionary for Regulatory Activities (MedDRA, version 11). AEs were summarized and presented by the number and percentage of patients with at least one AE of a specific PT by treatment arm. If one patient experienced one specific AE several times, the highest severity grade was considered for AE summary and for analysis. The comparison between ASAQ and AL arms in the main tolerability trial (Study-T) was performed with focus on a set of pre-selected AEs or pooled AEs of specific interest. Chi-square- or Fisher’s Exact test were used for comparisons between treatment groups, with a 5% significance level (two-tailed). No adjustment for multiple testing was made. Summary statistics (mean, standard deviation (SD)) at baseline and follow-up visit(s) were displayed for Hb (as measured by FBC), AST or ALT, neutrophil- and eosinophil counts. The highest follow-up severity grades *versus* baseline grades were displayed in shift tables for all patients for “AST or ALT increased”, or “neutropaenia”. All analyses were performed with STATA 10.1 (Stata Corp, Texas, USA).

## Results

### Inclusion and baseline parameters

**Figure 1:**
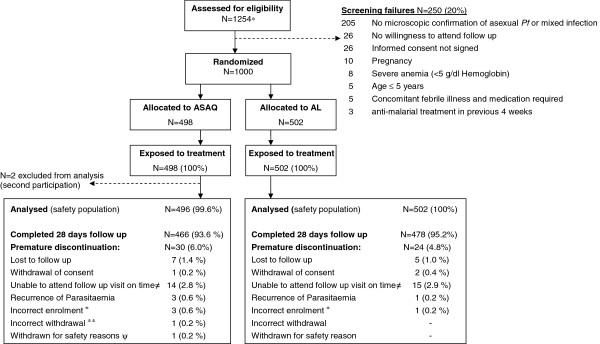
Trial profile (study-T, > 5 years).* Prescreened for positive Paracheck®, age >5 years, no signs of severe illness, willingness to attend the screening for study participation. ± Patient did not meet the inclusion criteria “asexual *P*. *falciparum* malaria parasites by blood smear” (had no asexual parasites on day 0), therefore withdrawn on day 0. ^± ±^ Accidentally discontinued on day 7 (presence of *P*. *falciparum* gametocytes by blood smear initially misinterpreted as failure). ψ Withdrawn on day 1 due to underlying severe hepatitis (positive for Hepatitis B s-antigen and anti-HBV core-antibody, indicating probable acute hepatitis at baseline).

**Figure 2:**
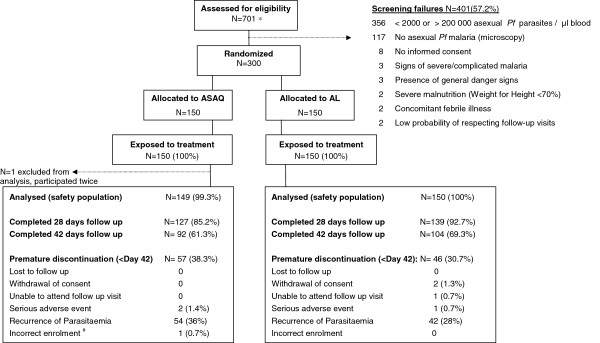
**Trial profile of (Study-E, 6–59 months).** * prescreened for positive Paracheck®, age 6–59 months, no signs of severe illness, willingness to attend the screening for study participation; ^#^ patient did not meet the inclusion criteria “asexual parasites density <2,000 or >200,000/μl blood” (parasite load of 415,082 trophozoites/μl blood), withdrawn on day 0.

In Study-T 1,000 patients were randomized, 498 allocated to the ASAQ arm and 502 to the AL arm (Figure 1). Two patients participated twice to the study. Their second participation was not included in the safety analysis (ASAQ arm, few mild or moderate blood system AEs were recorded at second participation). Most patients completed 28 days follow-up. One child (10 year old female, ASAQ arm) was withdrawn on day 1 due to underlying severe hepatitis with grade 3 increased ALT, grade 2 increased AST, grade 2 vomiting, and grade 1 fatigue and fever on day 0, and not improving on day 1. The patient was commenced on artesunate monotherapy. Retrospective day 0 serum testing indicated hepatitis B virus (HBV) infection with a probable recent or active hepatitis at baseline (HBV sAg and anti-HBVc IgM positive). In Study-E, 150 patients were randomized to each study arm (Figure 2). One child participated twice to the study. The second participation was not included in the safety analysis (ASAQ arm, mild blood system disorder AEs were recorded at second participation). In both studies, baseline characteristics were similar between treatment arms (Table 1).

**Table 1:** Patient characteristics at inclusion, safety population (both studies)

	**Study-T (> 5 years)**		**Study-E (6-59 months)**	
	**ASAQ**	**AL**	**ASAQ**	**AL**
	**N = 496**	**N = 502**	**N = 149**	**N = 150**
**Demographics and parasite load**				
Sex (male), N (%)	246 (49.6)	248 (50)	86 (57.7)	89 (59.3)
Mean age (in years, Study-T; in months, Study-E) (SD)	17.3 (12.1)	16.5 (10.6)	37.2 (13.7)	37 (13.6)
Mean weight in kg (SD)	38.3 (15.5)	38.2 (15.5)	12.8 (2.5)	12.9 (2.6)
Geometric mean *P. falciparum* trophozoite density (/μl), (range)*	624	558	20,020	19,151
	(16–355,623)	(16–285,011)	(2,016-415,082)	(2,000-194,127)
*P. falciparum* and *P. malariae* mixed infection, N (%)	7 (1.4)	9 (1.8)	-	-
**Clinical- and laboratory signs, patients N (%) (any severity grade)**				
Fever / history of fever in past 48 hrs	491 (99.0)	497 (99.0)	149 (100)	150 (100)
Fatigue	75 (15.1)	76 (15.1)	16 (10.7)	12 (8.0)
Vomiting	48 (9.7)	49 (9.8)	29 (19.5)	21 (14.0)
Diarrhoea	8 (1.6)	11 (2.2)	14 (9.4)	13 (8.7)
Splenomegaly	24 (4.8)	20 (4.0)	21 (14.1)	19 (12.7)
Hepatomegaly	6 (1.2)	7 (1.4)	2 (1.3)	4 (2.7)
Anaemia total	82 (16.5)	80 (15.9)	25 (16.8)	20 (13.3)
Anaemia ≥ grade 3	2 (0.4)	2 (0.4)	10 (6.7)	12 (8.0)
Neutropaenia ^1^	78 (15.7)	81 (16.1)	21 (14.4)	16 (10.8)
Thrombocytopaenia ^2^	26 (5.2)	12 (2.4)	6 (4.0)	5 (3.3)
AST increased	38 (8.6)	35 (7.7)	9 (6.1)	10 (6.7)
ALT increased	21 (4.6)	22 (6.2)	3 (2.0)	5 (3.3)
Serum creatinine increased	8 (1.9)	15 (3.6)	1 (0.7)	1 (0.7)
Serum bilirubine (total) increased	12 (2.9)	17 (4.0)	11 (8.0)	13 (9.4)

*SD* = standard deviation, *AST* = aspartate aminotransferase, *ALT* = alanine aminotransferase.

^1^Neutropaenia: (cells/μl): <12 years: ≤1,200, ≥12 years: ≤1,500.

^2^Thrombocytopaenia: (cells/μl): <12 years: ≤75,000, ≥12 years: ≤99,999.

Anaemia: Hb: (g/dl): <12 years: ≤10.9, ≥12 years: ≤10.5.

AST or ALT increased: <12 years: ≤ 4.9 × ULN, **≥**12 years: ≤2.5 × ULN.

Creatinine increased: <2 years: ≤ 0.8 × ULN, 2- <12 years: ≤1.0, ≥12 years: ≤1.5 × ULN; Bilirubin increased: <12 years or ≥12 years: ≤1.5 × ULN.

Study-T, day 0 measures available:

Hb (Beckman FBC): ASAQ: N = 495, AL: N = 501.

Neutrophil counts: ASAQ: N = 487, AL: N = 496.

Thrombocyte counts: ASAQ: N = 495, AL: N = 501.

AST: ASAQ: N = 441, AL: N = 452.

(Day 0 AST values retrospectively assessed from frozen serum samples: N = 100 ASAQ arm, N = 100 AL arm were included in this table).

ALT: ASAQ: N = 341, AL: N = 352.

Creatinine: ASAQ: N = 412, AL: N = 421 (retrospective from frozen serum).

Bilirubine: ASAQ: N = 413, AL: N = 422 (retrospective from frozen serum).

Study-E, day 0 measures available: Hb (Beckman FBC): ASAQ: N = 146, AL: N = 148, Neutrophil count: ASAQ: N = 139, AL: N = 140.

Thrombocyte count: ASAQ: N = 146, AL: N = 148.

AST: ASAQ: N = 148, AL: N = 150; ALT: ASAQ: N = 148, AL: N = 150.

Creatinine: ASAQ: N = 137, AL: N = 138 (retrospective from frozen serum).

Bilirubine: ASAQ: N = 137, AL: N = 138 (retrospective from frozen serum).

### Tolerability in patients aged over five years (Study-T)

Most patients experienced at least one AE (Table 2). These were mainly of mild or moderate severity, and none led to treatment discontinuation or withdrawal. Fatigue was the most frequently reported clinical AE (ASAQ: 200 (39.8%); AL: 81 (16.3%); p < 0.001) (Tables 2, 3 and 4). Gastrointestinal AEs, abdominal pain, anorexia or vomiting were also common in both arms. Two patients vomited after treatment intake and doses were re-administered and well tolerated (ASAQ arm). Frequent laboratory AEs included mild or moderate eosinophilia, neutropaenia, or anaemia in both arms (Table 2). Overall, mean Hb, AST or ALT values, neutrophil and platelet counts remained normal during follow up (see Additional file 1). An increase in eosinophil counts between day 0 and day 28 above normal range occurred in both arms (Additional file 1). Liver abnormalities were almost exclusively asymp-tomatic and mildly increased AST and/or ALT values (Tables 2, 3 and 4, Additional file 1). No dystonia or other severe neurotoxicities were reported (Tables 2 and 3). The few severe AEs in both arms were mainly asymptomatic laboratory events. Among these were three severe neutropaenia AEs (definition for severe: <400 cells/μl in children three months to 12 years, <750 cells/μl in ≥12 years [19]) (Tables 3 and 5), and two severe thrombocytopaenia AEs (both patients had elevated thrombocyte counts at baseline) (Table 3). Four patients had severe increased AST AEs, one patient in combination with a severe increased ALT (Table 3). Two of these patients (one in each arm) had normal or mildly increased liver-function-tests (LFTs) at baseline and their LFTs improved to grade 1 or 2 during post-study follow up. They were both non-reactive for Hepatitis B, C or E virus tests. The other two patients had already severely increased AST and/or ALT at baseline and experienced further increase without clinical manifestation during follow up (ASAQ arm). Both tested Hepatitis B s-antigen (HBV sAg)-positive. LFT values did not improve at post-study follow-up visits at 2 or 3 month after day 0. One patient presented on day 21 with symptomatic severe hepatitis AE (onset day 14, 8 year-old female, ASAQ arm) with cough, yellow eyes, dark yellow urination and grade 4 increased AST and ALT values and HBV sAg positive. The condition required no hospitalization. Clinical symptoms and elevated LFTs resolved about one month after diagnosis.

**Table 2:** Summary of adverse events recorded up to day 28, safety population (both studies)

	**Study-T (> 5 years)**		**Study-E (6-59 months)**	
**Patients N (%)**	**ASAQ**	**AL**	**ASAQ**	**AL**
	**N = 496**	**N = 502**	**N = 149**	**N = 150**
**AE summary**				
At least one AE (any grade)	457 (92.1)	453 (90.2)	141 (94.6)	132 (88.0)
At least one AE ≥ grade 3	17 (3.4)	8 (1.6)	6 (4.0)	4 (2.7)
Serious AE (SAE)	1 (0.2) *	0 (0)	2 (1.3)	1 (0.7)
AE leading to treatment discontinuation	0	0	0	1 (0.7)
**Common clinical- or laboratory AEs (≥5% in at least one study arm, any severity grade) #**				
Fatigue **	200 (39.8)	81 (16.3)	43 (28.9)	20 (13.3)
Headache	52 (10.5)	42 (8.4)	*2 (1.3)*	*2 (1.3)*
AE Vomiting (any)	35 (7.1)	8 (1.6)	16 (10.7)	10 (6.7)
AE Vomiting (after drug intake)	*2 (0.4)*	*0*	9 (6.0)	4 (2.7)
Abdominal pain	29 (5.8)	18 (3.6)	*3 (2.0)*	*4 (2.7)*
Anorexia	29 (5.8)	7 (1.4)	*2 (1.3)*	*2 (1.3)*
Cough	*-*	*-*	28 (18.8)	21 (14.0)
Diarrhoea	*20 (4.0)*	*14 (2.8)*	14 (9.4)	14 (9.3)
Eosinophilia ≠	144 (29.0)	185 (36.9)	6 (4.0)	9 (6.0)
Neutropaenia	94 (19.0)	110 (21.9)	6 (4.0)	8 (5.3)
Leukopaenia	37 (7.5)	29 (5.8)	10 (6.7)	3 (2.0)
Leukocytosis	21 (4.2)	44 (8.8)	10 (6.7)	11 (7.3)
Anaemia	74 (14.9)	49 (9.8)	34 (22.8)	23 (15.3)
AST increased	*17 (3.4)*	*21 (4.2)*	8 (5.4)	12 (8.0)
ALT increased	*11 (2.2)*	*17 (3.4)*	10 (6.7)	12 (8.0)

*AE* = adverse event, *AST* = aspartate aminotransferase, *ALT* = alanine aminotransferase.

**#** This section provides a summary of the most common AEs recorded (≥5%, any treatment arm), regardless of the severity or relationship to the study drug.

*In cursive letters*: ≥5% in at least one arm of any of the two parallel studies, respectively.

* SAE spontaneous abortion (ASAQ arm, Study-T).

** Pooled were reported events of “weakness”, “fatigue” and “asthenia”.

≠ AE recorded if above normal range (0.2-2.0 < 6 years; 0.3-0.8 × 10^3^/μL ≥ 6 - <12 years, 0.04 -0.4 × 10^3^/μL ≥12 years).

**Table 3:** Summary of severe adverse events recorded up to day 28, safety population (both studies)

	**Study-T (>5 years)**		**Study-E (6–59 months)**	
**Patients N (%)**	**ASAQ**	**AL**	**ASAQ**	**AL**
	**N = 496**	**N = 502**	**N = 149**	**N = 150**
**AEs with severity grade ≥ 3**				
Hepatitis	1 (0.2)	0	-	-
Stomatitis	-	-	0	1 (0.7)
Vomiting #	-	-	0	1 (0.7)
Malaria #	-	-	1 (0.7)	0
Pneumonia #	-	-	1 (0.7)	0
Splenomegaly	7 (1.4)	1 (0.2)	-	-
Thrombocytopenia	2 (0.4)	0	4 (2.7)	1 (0.7)
Leukocytosis	2 (0.4)	4 (0.8)	-	-
Neutropaenia	2 (0.4)	1 (0.2)	-	-
Anaemia	1 (0.2)	0	3 (2.0)	1 (0.7)
AST increased	3 (0.6)	1 (0.2)	-	-
ALT increased	1 (0.2)	0	-	-
Hepatomegaly	0	1 (0.2)	-	-

*AE* adverse event, *AST* aspartate aminotransferase, *ALT* alanine aminotransferase.

# These severe AEs were also reported as serious AEs (Study-E).

**Table 4:** Patients with at least one AE of specific interest, safety population, Study-T (>5 years)

**Patients N (%)**	**ASAQ**	**AL**	**p-value**
	**N = 496**	**N = 502**	
AEs “possibly related to study drug”	382 (77.0)	348 (69.3)	0.006
AEs ≥ grade 3	17 (3.4)	8 (1.6)	0.064
Fatigue (pooled) **	200 (39.8)	81 (16.3)	<0.001
Abdominal pain	29 (5.8)	18 (3.6)	0.091
Diarrhoea	20 (4.0)	14 (2.8)	0.279
Vomiting	35 (7.1)	8 (1.6)	<0.001
Vomiting after drug intake	2 (0.4)	0	0.247*
Nausea	16 (3.2)	5 (1.0)	0.015*
Hepatotoxicity ***	23 (4.6)	31 (6.3)	0.283
Rash	4 (0.8)	0	0.061*
Itching	0	0	-
Abnormal movements (Dystonia)	0	0	-
Neutropaenia	94 (19.0)	110 (21.9)	0.246
Anaemia	74 (14.9)	49 (9.8)	0.013
Thrombocytopaenia	4 (0.8)	0	0.061*

*AE* adverse event; * Fisher exact test; ** Frequencies of reported PTs “weakness”, “fatigue” and “asthenia” were pooled; *** defined as any or combination of the following AEs: hepatitis, hepatomegaly, AST and/or ALT increased, jaundice.

**Table 5:** Neutropaenia day 0 severity grade compared to maximum severity grade at follow up, safety population

**Study-T**		**ASAQ**	**AL**
**(>5 years)**		N = 482*	N = 490*
**Neutropenia grade at day 0**	**Maximum Neutropenia grade after day 0**	**Patients N (%)**	
0	0	332 (68.9)	325 (66.3)
1	0	33 (6.8)	25 (5.1)
2	0	2 (0.4)	2 (0.4)
*missing*	0	3 (0.6)	3 (0.6)
0	1	59 (12.2)	73 (14.9)
1	1	28 (5.8)	31 (6.3)
2	1	3 (0.6)	7 (1.4)
3	1	0 (0.0)	2 (0.4)
*missing*	1	4 (0.8)	3 (0.6)
0	2	6 (1.2)	7 (1.4)
1	2	6 (1.2)	5 (1.0)
2	2	6 (1.2)	2 (0.4)
3	2	0 (0.0)	1 (0.2)
1	3	2 (0.4)	1 (0.2)
3	3	1 (0.2)	0 (0.0)
Any worsening after baseline		73 (15.1)	86 (17.6)
**Study-E**		**ASAQ**	**AL**
**(6-59 months)**		N = 142*	N = 143*
**Neutropenia grade at day 0**	**Maximum Neutropenia grade after day 0**	**Patients N (%)**	
0	0	121 (85.2)	125 (87.4)
1	0	3 (2.1)	3 (2.1)
*missing*	0	9 (6.3)	7 (4.9)
0	1	6 (4.2)	6 (4.2)
1	1	3 (2.1)	2 (1.4)
Any worsening after baseline		6 (4.2)	6 (4.2)

* Shift tables were created for all patients with at least one neutrophil count available after day 0.

**Figure 3:**
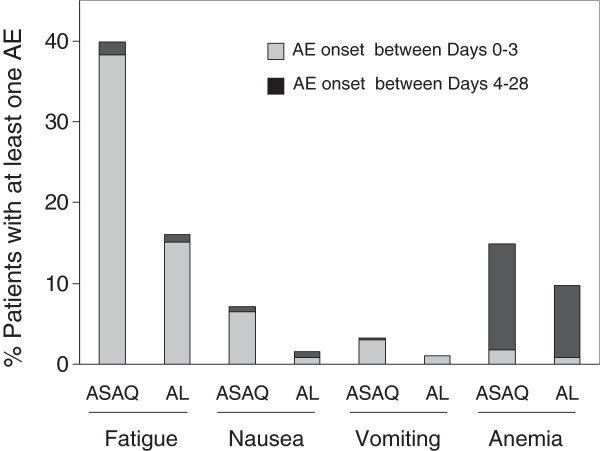
**Onset period of specific AEs of interest(Study-T, >5 years).** AE = adverse event. Depicted are the percentage of patients who had at least on AE fatigue, vomiting, nausea or anaemia, respectively, classified by event onset time period; i e, between day 0 after treatment intake and day 3, or between day 4 and day 28, respectively, by treatment arm. Safety population.

The one patient with severe anaemia AE (ASAQ arm, Hb 7.9 g/dl) had grade 2 anaemia at baseline (8.0 g/dl), and completely recovered during follow up. One serious AE (SAE), spontaneous abortion, was recorded for a 15 year-old female patient (ASAQ arm) with a positive day 28 β-HCG urine-pregnancy test (test negative at inclusion) who reported symptoms of vaginal discharge and lower abdominal pain (with onset nine days earlier). A sexually transmitted infection was suspected and treated with standard antibiotics and symptoms subsided. A post-study follow up urine pregnancy test (day 40) was negative, and spontaneous abortion diagnosed, declared as - serious AE “possibly related to study drug”. A retrospective blood β-HCG test on day 0 serum was positive (601.2 international units per litre (IU/L)), indicating early pregnancy at inclusion. The direct comparison between treatment arms on a set of pre-selected AEs of specific interest revealed significantly higher frequencies of mild or moderate fatigue, vomiting, anaemia and nausea in the ASAQ arm (Table 4). These were early events that largely occurred before day 4, with the exception of anaemia (Figure 3), and largely resolved before day 28 (fatigue AE both arms: 97% completely recovered; vomiting AE: ASAQ: 100%, AL: 87.5% completely recovered; nausea AE: both arms 100% completely recovered; anaemia AE: ASAQ: 79% and AL: 74% completely recovered).

### Tolerability in patients aged six to 59 months (Study-E)

Most children experienced at least one AE. Mild or moderate fatigue, cough, vomiting and diarrhoea were most common clinical AEs in both arms (Table 2). Mean Hb values improved from mild anaemia on days 0, 7 to normal on day 28, and mean neutrophil counts, and AST and ALT values remained within normal range during follow up (see Additional file 1). The mean eosinophil count increased two-fold up to day 28 in both arms (within normal range). Among laboratory AEs, mild or moderate anaemia and AST/or ALT increased were frequent. The only clinical hepatotoxicity was mild jaundice in one patient (AL arm, outcome recovered, not tabulated). Severe AEs were infrequent and included anaemia, thrombocytopaenia, and four clinical AEs: stomatitis (AL), severe malaria (ASAQ, day 28 *P*. *falciparum* malaria re-infection confirmed by parasite genotyping), severe pneumonia (ASAQ), severe vomiting (AL, repeated vomiting of the study drug leading to study discontinuation) (Table 3). The three later events were also declared as serious AEs since hospitalization was required. All serious AEs resolved without sequelae.

### Blood concentrations of amodiaquine, desethyl-amodiaquine , lumefantine among patients with certain adverse events (Study-T and Study-E)

AQ and DEAQ blood concentrations (ASAQ arm) and LF blood concentrations (AL arm) were assessed from day 0 and day 7 blood spots. In Study-T, AQ intake shortly before blood sampling (protocol violation) was indicated for one patient with quantifiable AQ on day 0, for one patient with high DEAQ blood concentration (>200 ng/ml) on day 0, and for four patients with quantifiable AQ on day 7. Four patients had day 7 DEAQ values <100 ng/ml, suggesting insufficient intake or malabsorption. One hundred patients in the ASAQ arm had low day 0 DEAQ concentrations (median 3.9 ng/ml), indicating AQ intake > four weeks before inclusion (no deviation). In the AL arm, 19 patients had non-quantifiable LF values on day 7 (<LOQ 200 ng/ml). Their day 7 values were set to LOQ/2 (100 ng/ml), since the LOQ was considered low.

Study-T: Median day 7 DEAQ and LF concentrations are depicted in Table 6. No differences in DEAQ or LF blood concentrations, respectively, were identified between patients with or without selected AEs of interest (Table 6). The exception was significantly higher day 7-DEAQ concentrations found among patients who experienced vomiting as an AE, than those without (p = 0.003).

**Table 6:** Day 7 DEAQ and LF blood concentrations by presence or absence of selected AEs, safety population, Study-T

**Study-T**	**ASAQ**			**AL**		
**(>5 years)**	**N = 496**			**N = 502**		
**AEs of interest**	**Patients**	**DEAQ blood concentration**	**p-value**	**Patients**	**LF blood concentration**	**p-value**
	n/N*	**[ng/ml] median (IQR)**	**	n/N*	**[ng/ml] median (IQR)**	**
All patients	421/496	466 (352,606)		447/502	505 (I 380,670)	
**Fatigue (pool)**						
No AE	250/296	466 (352,605)	p = 0.925	376/421	504 (381,659)	p = 0.549
AE	171/200	466 (352,606)		71/81	519 (374,709)	
**Vomiting**						
No AE	393/461	461 (345,603)	p = 0.003	440/494	505 (381,670)	p = 0.711
AE	28/35	559 (467, 705)		7/8	463 (277, 828)	
**Nausea**						
No AE	407/480	466 (352, 605)	p = 0.754	443/497	505 (381, 670)	p = 0.111
AE	14/16	457 (387, 648)		4/5	245 (100, 549)	
**Anaemia**						
No AE	350/422	462 (352, 604)	p = 0.162	403/453	505 (381, 701)	p = 0.745
AE	71/74	517 (345, 677)		44/49	508 (374, 616)	
**Hepatotoxicity**						
No AE	402/473	465 (352, 606)	p = 0.512	418/471	506 (378, 681)	p = 0.882
AE	19/23	485 (407, 586)		29/31	477 (416, 659)	

* n/N = number of blood samples available and quantifiable (n) among total patients with or without AE (N); ** Two-sample Wilcoxon rank-sum (Mann–Whitney) test.

Study-E: very recent AQ intake was indicated for six children who had quantifiable AQ concentrations on day 0 (range: 3.6–34 ng/ml, n = 3 with detectable DEAQ (>200 ng/ml)) and five children with quantifiable AQ on day 7 (range: 2.6-4.2 ng/ml) (protocol violation). Three children had non-quantifiable DEAQ on day 7 (<2.5 ng/ml), suggesting malabsorption. In the AL arm, 33 patients had non-quantifiable day 7 LF concentrations (<LOQ, 200 ng/ml), which were set as LOQ/2. The median day 7 DEAQ blood concentration was significantly higher in children with AE fatigue (p = 0.004) than in children without (ASAQ arm, Table 7). The median day 7 LF concentrations were significantly higher in children with anaemia AE than without (AL arm, p = 0.03), and significantly lower in children with any hepatotoxicity AE compared to children without (p = 0.049) Table 7. Results were comparable when patients with recent AQ intake (day 0, day 7), low day 7 DEAQ or non-quantifiable day 7 LF values were excluded from the analyses (both studies), except significantly higher day 7 DEAQ values in patients with any hepatotoxicity AE (Study-E) when patients with deviations were excluded (p = 0.036) (Table 7 footnote).

**Table 7:** Day 7 DEAQ and LF blood concentrations by presence or absence of selected AEs, safety population, Study-E

**Study-E**	**ASAQ**			**AL**		
**(6–59 months)**	**N = 149**			**N = 150**		
**AEs of interest**	**Patients**	**DEAQ blood concentration**	**p-value**	**Patients**	**LF blood concentration**	**p-value**
	n/N*	**[ng/ml] median (IQR)**	**	n/N*	**[ng/ml] median (IQR)**	**
All patients	137/149	423 (314,602)		139/150	310 (200,447)	
**Fatigue (pool)**						
No AE	93/105	396 (302,529)	p = 0.004	122/130	332 (200,447)	p = 0.332
AE	44/44	501 (354,794)		17/20	256 (100,344)	
**Vomiting**						
No AE	122/133	435 (314,602)	p = 0.725	130/140	313 (200,447)	p = 0.428
AE	15/16	366 (277,743)		9/10	256 (100,329)	
**Nausea**						
No AE	137/149	423 (314,602)	*na*	138/149	310 (200,447)	*na*
AE	0/0	-		1/1	256	
**Anaemia**						
No AE	105/115	423 (310,554)	p = 0.455	116/127	305 (150,431)	p = 0.030
AE	32/34	443 (332,739)		23/23	380 (262,566)	
**Hepatotoxicity**						
No AE	125/136	416 (311,554)	p = 0.065#	121/132	329 (217,457)	p = 0.049
AE	12/13	569 (354,934)		18/18	150 (100,409)	

AE = adverse event; * n/N = number of blood samples available and quantifiable (n) among total patients with or without AE (N); ** Two-sample Wilcoxon rank-sum (Mann–Whitney) test; # When patients with deviations (quantifiable AQ on day 0 or day 7) were excluded from this comparative analysis (n = 6): p = 0.036.

### Viral hepatitis serology at baseline

Retrospective viral hepatitis serology on baseline serum samples from both studies revealed in the > five years population (Study-T) around 15% HBV prevalence (HBV sAg positive), among these about 4% with a probable (recent) active hepatitis (IgM anti-HBc positive). A total of 4% of included patients were identified as HCV carriers (Study-T). Among children six to 59 months (Study-E), 12% were HBV carriers (HBV sAg positive), and among these about 5% were also IgM anti-HBc positive. A total of 4% were HCV carriers (Study-E) (Table 8).

**Table 8:** Baseline viral hepatitis serology, safety population

**Patients n/N (%)**			
	**ASAQ**	**AL**	
**Study-T (>5 years)**	**N = 496**	**N = 502**	**Total**
HBV sAg positive	73/457 (15.9)	70/467 (14.9)	143/924 (15.5)
HBV anti-HBVc positive #	4/73 (5.5)	2/70 (2.8)	6/143 (4.2)
HCV antibody positive	18/455 (4.0)	19/466(4.1)	37/921 (4.0)
HEV anti-IgM positive*	0/33 (0)	0/34(0)	0/67 (0)
	**ASAQ**	**AL**	
**Study-E (6-59 months)**	**N = 149**	**N = 150**	**Total**
HBV sAg positive	18/148 (12.1)	18/150 (12.0)	36/298 (12.1)
HBV anti-HBVc positive #	1/18 (5.5)	1/18 (5.5)	2/36 (5.5)
HCV antibody positive	7/147 (4.8)	4/148 (2.7)	11/295 (3.7)
HEV anti-IgM positive*	0/5 (0)	0/7 (0)	0/12 (0)

*HBV* Hepatitis B Virus, *HCV* Hepatitis C Virus, *HEV* Hepatitis E Virus, *Ag* antigen.

Displayed are percentages of patients who tested positive among those with available test results.

# anti-HBVc was assessed for HBV sAg positive patients.

* HEV anti-IgM was assessed for patients with elevated AST or ALT (values 2× upper limit normal range) at any point during study participation, and/or any AE “hepatotoxicity” among Study-T participants.

## Discussion

The tolerability profile of two widely used fixed-dose ACTs, ASAQ and AL, for the treatment of uncomplicated *P*. *falciparum* malaria, was assessed in a trial of 1,000 patients over five years old, an age group not frequently studied in the past and able to express symptoms. The findings were complemented with safety data collected in a parallel trial from 300 children under five years. In both studies, ASAQ and AL were very well tolerated and highly efficacious (>90% genotyping-adjusted cure rates, [13]. Clinical- or laboratory adverse events (AEs) reported in both studies corresponded closely to abnormalities seen in malaria infection itself, and were overall consistent with recent findings. Severe AEs were few and mostly laboratory abnormalities, which did not manifest clinically. Four SAEs were reported. The only SAE in Study-T was a spontaneous abortion in a 15 years-old patient, for which a potential drug relationship could not be excluded, though abortion is also known to occur frequently in malaria [20]. Among children < 5 years (Study-E), SAEs were one event of pneumonia and one case of severe malaria (ASAQ arm), and one severe vomiting after drug intake (AL arm). All three SAEs were completely resolved.

Neutropaenia and anaemia AE were frequent in both studies, with few severe events. The overall effects of malaria infection or treatment(s) on Hb and neutrophil counts in both age-groups were minimal, in line with recent data [3,21,22]. Eosinophilia was observed in both studies, likely to reflect helminth infections [23]. Severe hepatotoxicity, neutropaenia, dystonia, events previously reported with high-dose AQ [5-8], were of no specific concern in the present studies, in line with recent trials on AS + AQ and/or AL tolerability [3,4,11,24-26]. Retrospective serology on day 0 serum samples indicated a high prevalence of chronic Hepatitis B infection among participants of the tolerability trial (15%). Three of the HBV-positive patients experienced grade 3 or 4 increased AST and/or ALT AEs (ASAQ arm). Two had already entered the trial with asymptomatic but severely elevated LTF(s) and developed no clinical manifestation during study follow-up. One HBV-positive patient experienced a transient symptomatic hepatitis during the study. No specific medical intervention was indicated and clinical- and laboratory signs resolved during post-study follow up. In the second study among children under five years, the prevalence of indicated chronic HBV infection was also high (12%). No severe hepatotoxicity AEs were recorded in this second trial. Taken together, our findings may suggest that ASAQ and AL are safe and also overall well tolerated among patients with chronic viral hepatitis, a condition that is very common in tropic settings where malaria is endemic. It should be noted however that the trials were not powered to detect rare events. Further surveillance in this specific sub-population may be required, including repeated treatments in highly endemic settings. Recent studies also indicated a potential increase of adverse events among patients co-infected and treated for both malaria and HIV [27,28]. Information on the HIV-serostatus of patients in the present studies was not recorded, but the estimated HIV prevalence in Liberia is relatively low (1%) [29]. None of the participants reported antiretroviral treatments among recorded concomitant medications.

A comparative analysis on the frequency of patients presenting with adverse events of specific interest was performed in the main tolerability trial (Study-T). The only difference identified between treatment arms were higher frequencies of mild or moderate AEs fatigue, vomiting, nausea, and anaemia recorded in the ASAQ arm. The clinical events were mostly early onset (≤day 3), did not affect treatment intake, and were largely resolved before day 28.

Mild or moderate fatigue AEs were very frequently reported in both treatment arms, with almost twice as many patients in the ASAQ arm than in the AL arm (39.8% versus 16.3%, p < 0.001). Asthenia is considered among common undesirable effects of ASAQ Winthrop (SmPC ASAQ Winthrop®), similar to what is noted for AL [3]. No differences in fatigue or asthenia between AS + AQ and AL arms were reported in four recent studies [11,24,30,31]. In the present trials, fatigue was among the symptoms prompted by the physicians during clinical examination, and some degree of over-reporting of more subjective symptoms such as fatigue cannot be fully excluded in an open-label trial.

Among gastrointestinal AEs, mild or moderate vomiting AE was about four times more frequently reported in the ASAQ arm. The incidence of early vomiting immediately after drug intake though was reassuringly low in both study arms. Recent trials conducted in Ghana, Senegal and Ivory Coast, respectively, also reported nausea and vomiting AEs at higher frequency in the AS + AQ arm when compared to AL [24,30]. No differences in gastro-intestinal AEs were reported in three other trials [11,24,31]. The impact of such mild or moderate clinical symptoms on real life treatment adherence and effectiveness would be worthy of further study, taking into account also other factors such as concomitant food intake, tablet burden and dosing schedule. In the present studies, ASAQ was not administered with food, since a high fat meal may modify the bioavailability of AS and AQ [32]. Mild or moderate anemia AEs were also more frequent in the ASAQ arm, similar to one recent study [30]. Mean Hb values remained within the normal range in both treatment arms, indicating that a potential effect of the treatment(s) on haemoglobin value was overall very moderate. A recent review which summarized the findings of nine trials in African Countries on AL versus AS + AQ however did not identify differences in anemia AEs [21].

The bioavailability of the non-artemisinin partner compound of ACTs may also affect tolerability. Among patients over five years (Study-T), the day 7 DEAQ-blood concentration was significantly higher for patients with vomiting AE compared to patients without vomiting AE. Among children under five years (Study-E), the findings were different. Higher day 7-DEAQ values were identified among children with fatigue or hepatotoxicity AE than without, and a higher day 7-LF concentration was found among children with anaemia AE than without. The relevance of these associations is uncertain. Further studies and pooled data analyses are needed to better interpret the relationships of blood concentrations, dosage, and tolerability.

Safety and tolerability monitoring of ASAQ FDC and all other ACTs should continue in a standardised manner. Pharmacovigilance networks are not implemented in most settings were ACTs are routinely used. Post-marketing information on safety and tolerability thus relies on individual studies. The need of standardized guidelines for adverse event monitoring of ACTs was recently emphasized [33,34]. More information on the tolerability of ACT specifically for patients suffering from chronic (viral) hepatitis, and/or HIV infection would also be of use.

## Conclusions

These findings confirmed the good tolerability of ASAQ FDC (Winthrop®) and AL (Coartem®) in patients of all ages in Liberia. Hepatotoxicity, neutropenia or dystonia were of no specific concern in the present studies. Tolerability monitoring of ASAQ FDC should continue, as for all widely used ACT.

## Competing interests

The authors declare that they have no competing interests.

## Authors’ contributions

BS: overall trial coordination, participation in study design, protocol and data analysis plan, study documents, writing of the manuscript. PV: field trial coordination, medical coordination, trial team supervision, supervision of data collection. EB: support study design, coordination of data management, data analysis plan, data analysis, revision of the manuscript. CM: field laboratory coordination, laboratory team supervision, - standard operating procedures, - data collection. RS: field coordination during study preparation, writing of administrative and clinical standard operating procedures, preparation of study site, staff training and trial implementation. LP: data management, support data analysis plan, data analysis, revision of the manuscript. MD and JB: coordination and interpretation of the malaria parasite genotyping. TS: support for field trial coordination, team supervision and data collection. YMZ and JJJ: technical support to all study steps, participation in study implementation and training. EC: support study initiation, participation in study design, technical support. AB: baseline biochemistry (AST, ALT) for sample subset and baseline creatinine (all patients). MB: baseline viral hepatitis serology. VJ: development and conduct of blood concentration analyses of artemisinin-partner compounds. GC: technical advise to protocol development, study preparation, support field training and study conduct, review of study documents, coordination of study monitors and data monitoring committee, revision of the manuscript. JRK: study initiation, technical and scientific advice to study protocol, study preparation and trial conduct, review of study documents, revision of the manuscript. EAA: study initiation, study design and protocol, scientific and medical advise to all steps of the study, revision of study documents, support data analysis plan, revision of the manuscript. PJG: study initiation, study design and protocol, scientific and medical advise to all steps of the study, review of study documents, medical review of adverse events, support data analysis plan, revision of the manuscript. All authors read and approved the manuscript.

## Supplementary Material

Additional file 1 Mean liver enzyme (AST/ALT) and blood cell parameter values on Days 0, 7 and 28, and change from baseline, respectively, by treatment group – Safety population, both studies. Click here for file
