# Supplementary material for: Tolerability and safety of artesunate-amodiaquine and artemether-lumefantrine fixed dose combinations for the treatment of uncomplicated Plasmodium falciparum malaria: two open-label, randomized trials in Nimba County, Liberia
Source: Malar J. 2013 Jul 17;12:250. doi: 10.1186/1475-2875-12-250 (PMC3728046; doi:10.1186/1475-2875-12-250)
Supplement: Additional file 1 — Mean liver enzyme (AST/ALT) and blood cell parameter values on Days 0, 7 and 28, and change from baseline, respectively, by treatment group – Safety population, both studies. [file 1475-2875-12-250-S1.doc]

Additional File 1 Mean liver enzyme (AST/ALT) and blood cell parameter values on Days 0, 7 and 28, and change from baseline, respectively, by treatment group – Safety population, both studies.

|  | **Study-T (>5 years)** | | **Study-E (6-59months)** | |
| --- | --- | --- | --- | --- |
|  | ASAQ | AL | ASAQ | AL |
| N, mean (sd) | N=496 | N=501 | N=149 | N=150 |
| **AST (IU/L)*** |  |  |  |  |
| Day0 | N=318 *  32.6 (23.9) | N=332*  31.4 (14.9) | N=127  21.4 (26.9) | N=139  25.1 (64.7) |
| Day28 | N=318  36.3 (39.3) | N=332  37.6 (31.7) | N=127  20.7 (15.3) | N=139  21.2 (10.8) |
| Change from Day0 | N=318  3.7 (25.2) | N=332  6.2 (27.5) | N=127  -0.7 (28.6) | N=139  -3.8 (65) |
| **ALT (IU/L)** |  |  |  |  |
| Day0 | N=318  22.2 (20.9) | N=332  21.8 (16) | N=127  47.6 (93.5) | N=139  48.9 (106.6) |
| Day28 | N=318  24.1 (43.3) | N=332  24.1 (22.2) | N=127  42.8 (23.9) | N=139  44.2 (14) |
| Change from Day0 at Day28 | N=318  1.8 (27.9) | N=332  2.3 (18.8) | N=127  -4.8 (91.5) | N=139  -4.6 (106.7) |
| **Hemoglobin (Beckman) (g/dL)** |  |  |  |  |
| Day0 | N=495  12.2 (1.5) | N=501  12.3 (1.5) | N=146  9.2 (1.6) | N=148  9.3 (1.5) |
| Day7 | N=417  11.7 (1.4) | N=426  11.9 (1.3) | N=141  9.4 (1.4) | N=141  9.8 (1.2) |
| Day28 | N=459  12.4 (1.3) | N=472  12.3 (1.3) | N=127  11.0 (1.1) | N=137  11.0 (1) |
| Change from Day0 at Day28 | N=459  0.2 (0.9) | N=472  0.1 (0.9) | N=127  +1.8 (1.4) | N=137  +1.6 (1.4) |

**Additional File 1** continued

| **Neutrophils (x 10^3^/µl )** |  |  |  |  |
| --- | --- | --- | --- | --- |
| Day0 | N=487  2.43 (1.56) | N=496  2.35 (1.24) | N=139  2.91 (1.48) | N=140  2.97 (1.63) |
| Day7 | N=403  2.57 (1.4) | N=421  2.35 (0.89) | N=125  3.32 (1.64) | N=127  3.1 (1.32) |
| Day28 | N=446  2.05 (0.87) | N=453  2.07 (0.92) | N=109  2.64 (1.23) | N=117  2.66 (1.28) |
| Change from Day0 at Day28 | N=446  -0.41 (1.65) | N=453  -0.3 (1.3) | N=109  -0.13 (1.74) | N=117  -0.11 (1.54) |
| **Thrombocytes (x 10^3^/µl )** |  |  |  |  |
| Day0 | N=495  216.8 (77.2) | N=501  223.5 (78.6) | N=146  185.6 (97.1) | N=148  199.1 (104.2) |
| Day7 | N=417  286.2 (84.2) | N=426  286.5 (80.8) | N=141  403 (141.1) | N=141  399.7 (127.2) |
| Day28 | N=459  248.3 (70.5 | N=472  258.2 (73.8) | N=127  308.7 (110.8) | N=137  323.5 (99.6) |
| Change from Day0 at Day28 | N=459  30.1 (61.9) | N=472  34 (58.9) | N=127  127.1 (125.5) | N=137  125.5 (119.8) |
| **Eosinophils (x 10^3^/µl)** | | | | |
| Day0 | N=487  0.53 (0.81) | N=496  0.53 (0.63) | N=139  0.23 (0.41) | N=140  0.26 (0.49) |
| Day7 | N=403  0.81 (0.97) | N=421  0.96 (0.94) | N=125  0.47 (0.62) | N=127  0.52 (0.77) |
| Day28 | N=446  1.03 (1.53) | N=453  1.71 (2.51) | N=109  0.49 (0.55) | N=117  0.6 (0.75) |
| Change from Day0 at Day28 | N=446  0.5 (1.44) | N=453  1.17 (2.31) | N=109  0.24 (0.44) | N=117  0.36 (0.65) |

AST = aspartate aminotransferase; ALT= alanine aminotransferase

Depicted are mean and sd of AST, ALT blood concentrations and blood mean values with sd of blood parameters hemoglobin, neutrophil-, thrombocyte and eosinophil counts on Days 0, 7, 28, and the mean change between Day0 and Day28.

* AST values which were obtained retrospectively (frozen serum) for patients who had no LFTs done at baseline (PAT IDs 1 - 306) were not pooled with above tabulated data (ASAQ arm: N=100, mean (sd): 36.1 (20.7) IU/L, AL arm: N=100, mean (sd): 33.9 L (10.4) IU/L)

Normal ranges of blood and biochemistry parameters:

^b^For age groups: ≤ 6 yrs; 7-12 yrs; > 12 yrs (female); > 12 yrs (male):

Hemoglobin (g/dl): 9.5 - 13.5; 11.5 - 15.5; 11.5-16.5 ; 13.0-18.0;

Thrombocytes (10^3^/μl): 150- 400; 150 - 400; 150- 500; 150 – 400;

Neutrophils (10^3^/μl): 1.5-7.0; 2.0- 6.0; 2.0 - 7.5; 2.0 -7.5;

Eosinophils (10^3^/μl): 0.2-2; 0.3- 0.8; 0.04-0.4; 0.04-0.4;

^a^ For the age-groups: < 1 year; 1-3 yrs; 4-6 yrs; 7-12 yrs; 13-17 yrs; > 17 yrs (female); > 17 yrs (male): AST (IU/L): 22-58; 24-59; 16-48; 16-44; 14-39; ≤ 33; ≤ 40;

ALT (IU/L): 6-56; 8-29; 6-29; 8-37; 8-37; ≤ 32; ≤ 41

^a^ Reflotron plus® product information and Reference values for children and adults, Roche, 2007 (Reference method: IFCC without pyridoxalphosphate, at 37°C).

^b^ Default Patient Ranges, Act 5 diff analyzer, Beckman Coulter.
